# Supplementary material for: Risk Factors for Hepatitis E Virus Infection and Eating Habits in Kidney Transplant Recipients
Source: Pathogens. 2023 Jun 20;12(6):850. doi: 10.3390/pathogens12060850 (PMC10301935; doi:10.3390/pathogens12060850)
Supplement: Supplementary file 1 [file pathogens-12-00850-s001.zip › pathogens-2420415-supplementary.pdf]

Supplementary Table 1. Patient characteristics by status for cyclosporine intake

Patient characteristics	CyA intake N=55 N (%)	No CyA intake N=255 N (%)	Statistical group difference P-value
Gender			
Male	33 (60.0)	154 (60.4)	**1.000**
Female	22 (40.0)	101 (39.6)	
Age			
Mean (y)	55.1 ± 13.6	53.6 ± 13.7	0.515
<40	10 (18.2)	47 (18.4)	1.000
40-60	27 (49.1)	127 (49.8)	1.000
>60	18 (32.7)	81 (31.8)	0.875
Renal disease			
Glomerulonephritis	20 (36.4)	66 (25.9)	0.135
Cystic kidney disease	7 (12.7)	44 (17.3)	0.548
Diabetic nephropathy	7 (12.7)	33 (12.9)	1.000
Hypoplastic kidneys	1 (1.8)	16 (6.3)	0.325
Other	9 (16.4)	41 (16.1)	1.000
Unknown	11 (20.0)	49 (19.2)	0.853
Co-Morbidities			
Hypertension	49 (89.1)	199 (78.0)	0.065
Diabetes mellitus	13 (23.6)	70 (27.5)	0.618
Arteriosclerosis	17 (30.9)	66 (25.9)	0.502
History of cancer	8 (14.5)	38 (14.9)	1.000
Chronic liver disease	16 (29.1)	44 (17.3)	0.059
Time after transplantation (m)	145 ± 97	80 ± 75	**<0.001**
Type of transplant			0.746
Kidney	50 (90.9)	227 (89.0)	
Pancreas-kidney	4 (7.3)	18 (7.1)	
other multi-visceral	1 (1.8)	10 (3.9)	
Type of donation			
Postmortem	45 (81.8)	184 (72.2)	0.128
Living donation	10 (18.2)	56 (22.0)	
ABOi living donation	0 (0.0)	15 (5.9)	
Re-transplant	5 (9.1)	32 (12.5)	0.647
Immunosuppressive regimen			
Mycophenolic acid	43 (78.2)	236 (91.5)	**0.005**
mTOR inhibitor	1 (1.8)	15 (5.9)	0.322
Steroid	37 (67.3)	206 (80.8)	**0.031**
Triple IS	30 (54.5)	191 (74.9)	**0.005**
Dual IS	25 (45.5)	64 (25.1)	**0.005**

Data were expressed as mean (±standard deviation) or numbers (n), CyA= cyclosporine, y=years, m=months, IS= immunosuppression.

Supplementary Table 2. KTR with HEV infection subdivided by status for allograft function.

**Patient characteristics**	**Creatine ≤ 1.4 mg/dL N= 26**	**Creatine > 1.4 mg/dL N= 33**	
Baseline creatinine (mg/dL)	1.1 ± 0.2	1.9 ± 0.6	<0.01
Baseline eGFR (mL/min)	69 ± 15	38 ± 13	<0.01
Baseline proteinuria (mg/gCrea)	258 ± 407	448 ± 640	0.137
Peak proteinuria (mg/gCrea)	452 ± 601	929 ± 1079	0.042
Rise of proteinuria, n (%)	6 (23.1%)	9 (27.2%)	0.538
Stable eGFR, n (%)	13 (50%)	13 (39.4%)	0.441

Data were expressed as mean (±standard deviation) or numbers (n), KTR= kidney transplant recipients, HEV= hepatitis E virus, eGFR= estimated glomerular filtration rate, Crea= creatinine.
